# Human Enteroviruses isolated during acute flaccid paralysis surveillance in Ghana: implications for the post eradication era

**Published:** 2012-07-16

**Authors:** John Kofi Odoom, Evangeline Obodai, Jacob Samson Barnor, Miriam Ashun, Jacob Arthur-Quarm, Mubarak Osei-Kwasi

**Affiliations:** 1 Department of Virology, Noguchi Memorial Institute for Medical Research, University of Ghana, Legon, Accra, Ghana

**Keywords:** Poliovirus, non-polio enterovirus, surveillance, acute flaccid paralysis, Ghana

## Abstract

**Introduction:**

Surveillance of acute flaccid surveillance (AFP) has been used world-wide to monitor the control and eradication of circulating wild polioviruses. The Polio Laboratory since its accreditation in 1996 has supported the Disease Surveillance Department for AFP surveillance. This study aims to isolate and characterize human enteroviruses from patients with AFP in Ghana.

**Method:**

Stool suspension was prepared from 308 samples received in 2009 from the surveillance activities throughout the country and inoculated on both RD and L20B cell lines. Isolates that showed growth on L20B were selected for real-time RT-PCR using degenerate and non-degenerate primers and probes. RD isolates were however characterized by microneutralisation technique with antisera pools from RIVM, The Netherlands and viruses that were untypable subjected to neutralization assay using antibodies specific for E71.

**Results:**

Of the 308 samples processed, 17 (5.5%) grew on both L20B and RD cells while 32 (10.4%) grew on RD only. All 28 isolates from L20B were characterized by rRT-PCR as Sabin-like polioviruses. No wild poliovirus or VDPV was found. However from the microneutralisation assay, six different enteroviruses were characterized. Among these, Coxsackie B viruses were most predominant followed by Echovirus. Three children from whom non-polio enteroviruses were isolated had residual paralysis while one child with VAPP found. The non-polio enteroviruses circulated throughout the country with the majority (20.7%) from Ashanti region.

**Conclusion:**

This study showed the absence of wild or vaccine-derived poliovirus circulation in the country. However, the detection of three non-polio enteroviruses and one Sabin-like poliovirus with residual paralysis call for continuous surveillance even in the post polio eradication era.

## Introduction

The global effort to eradicate polio is the largest public health initiative in history. One of the eradication strategies recommended by the World Health Organization (WHO) is surveillance and laboratory investigations of acute flaccid paralysis (AFP) which gives the laboratory a crucial role in ensuring that the initiative meets its objectives [1].

Human Enteroviruses (HEVs) are genus of the Picornaviridae family, subdivided into Polioviruses and non-polio enteroviruses. The non-polio enteroviruses are further divided into human enterovirus A (HEV-A), HEV-B, HEV-C and HEV-D [2]. Poliovirus has 3 serotypes 1-3, HEV-A 18, HEV-B 60, HEV-C 17, HEV-D 3 and other new HEVs 11. The new enteroviruses range from 68-109. Most poliovirus infections are asymptomatic, with clinical symptoms being observed in only 0.1 to 1% of infections. Non-polio enteroviral infections may often cause a wide spectrum of potentially serious human diseases such as common cold, aseptic meningitis, encephalitis, myocarditis, diabetes, diarrhea, rash, acute hemorrhagic conjunctivitis or acute flaccid paralysis [3–5].

Since polioviruses and some non-polio enteroviruses can cause acute flaccid paralysis, all suspect cases must undergo thorough virological investigation. For instance, Echovirus has been implicated in multiple human disease syndromes, including paralysis [6]. WHO reports revealed that from 1967 to 1970, paralysis was present in less than 1% of all patients with echovirus and Coxsackie virus infections [7]. Moreover, 12 patients with residual paralysis from 77 AFP cases due to Coxsackie viruses have been reported in Scotland from 1956 to 1973 [8]. Enterovirus 71 is a recognized cause of epidemic severe central nervous system disease in Southeast Asia.

Although majority of AFP cases are mild and transient, persistent muscle weakness or total paralysis or even death has been reported [9]. For this reason it is essential that stool specimens from every identified AFP case be subjected to thorough and systematic examination for the presence of wild poliovirus since missing virus in one case may mean that a thousand infections have been missed. However not all Poliovirus or NPEVs induced AFP end in residual paralysis, but outbreaks of infections with NPEVs are worldwide [10]. The presence of enterovirus in a clinical sample can be readily determined by applying the specimen to a proper cell culture system, or directly by molecular detection of the 5’-NTR conserved region of the viral genome. For serotyping, a neutralization test has traditionally been used [11].

Active surveillance for cases of acute flaccid paralysis (AFP), the most common clinical manifestation of paralytic poliomyelitis, with full laboratory support to detect poliovirus was formally established in Ghana in 1996. A number of performance indicators have been used to monitor the quality of AFP surveillance and the laboratory work is also subjected to a full accreditation process to assess the efficiency of isolation and characterization of poliovirus from AFP stool samples.

The impending eradication of poliovirus and some of the challenging links between enterovirus infections and emerging diseases currently faced by the eradication program present the possibility that poliomyelitis could emerge in the post eradication era. Here we report on the laboratory findings, isolation and characterisation, of all AFP cases to the Polio Laboratory in 2009.

## Methods

### AFP Surveillance

Surveillance Officers throughout the country collected stool samples from acute flaccid paralysis cases from children under 15 years between January and December 2009 according to WHO standards. Two faecal specimens per AFP case, 24 hours apart and within 14 days of the onset of paralysis were sent in cool boxes with ice packs to maintain the cold chain to the National Laboratory. A large number of data were collected with every AFP case. They included details of the patient such as clinical symptoms, age, sex, location and immunisation history as well as technical aspects that affect the quality of stool sampling and laboratory analysis. Samples collected were sent to reach the polio laboratory within 72 hours of shipment.

The polio laboratory is a key component of the surveillance process as it allows assessing if there is a cause-effect association between the presence of poliovirus and the clinical AFP symptoms showed by a patient, which may be due to a number of syndromes other than polio. Such virological information can be used to monitor the progress of polio eradication guiding public health interventions to correct any deficiencies or to prevent the spread of epidemics. The polio laboratory at the Department of Virology, Noguchi Memorial Institute for Medical Research, University of Ghana in Accra was strengthened in 1996 to support the polio surveillance activities. The laboratory is a Regional Reference Laboratory for Africa but currently serves Ghana and Togo. The Polio Laboratory process faecal samples and report findings to the National EPI and WHO within 14 days of receipt.

The Surveillance officers with the help of Paediatrician examine the limbs of the patient after 60-90 days of onset of paralysis for residual paralysis. The Polio Expert Committee, appointed by the Ghana Health Service in the mid 1990s, reviews all notifications of AFP, irrespective of age. Using virological classification, cases are classified as polio due to wild poliovirus, VDPV or VAPP; non-polio AFP; or non-AFP.

### Virus isolation and characterization

Samples were processed in the laboratory according to WHO manual 2004 [2]. Briefly, faecal specimens were pretreated with chloroform and antibiotics in PBS before being inoculated onto a healthy monolayer of RD and L20B in serum-free medium. The cells were seeded 48 hours earlier into culture tubes with growth medium (Eagle's MEM supplemented with 10% FCS). The monolayers were then observed daily for the characteristic enterovirus cytopathic effects (CPE) of rounded, refractile cells detaching from the surface of the tube. The tubes with CPE up to 75% were harvested and stored at -20 C. Samples that were negative after 7 days of incubation were repassaged onto fresh cells and observed for another 7 days. Samples that showed CPE on L20B were selected for Real-Time Reverse Transcriptase polymerase Chain Reaction (rRT-PCR). Moreover, any culture positive on RD cells but negative on L20B cells was re-passaged on L20B cells to exclude polioviruses [2]. Identification of isolates on RD cells was carried out by microneutralisation technique using antisera raised in horse against Coxsackie and echoviruses prepared by the National Institute of Public Health and the Environment (RIVM), Netherlands. Microneutralisation assay with E71 antiserum was carried out on the isolates that were untypable with the antiseraum pools.

### Data Analysis

The MS excel data base was imported into SPSS version 16 and analyzed. Univariable analysis of case investigation and administrative data by person, place and time were expressed as frequency distributions, percentages and charts.

### Ethical Issues

Waiver for ethical approval for the study was obtained from the Institutional Review Board of the Noguchi Memorial Institute for Medical Research. We protected the confidentiality of patients through use of codes.

## Results

Ghana continues to use oral polio vaccine (OPV) for polio immunization which include routine and National Immunisation Days (NIDs). Routine OPV3 immmunisation coverage which involves 4 doses of OPV was 89% in 2009. During NIDs and sub-NIDs, 2 doses of OPV are giving to children under 60 months irrespective of previous immunization history. In 2009, three rounds of house-house NIDs conducted recorded coverages of 99.7, 103.5 and 104.1 respectively (higher than 100% coverage due to under estimation of denominator).

### Virus isolation and characterization

Stool suspensions from a total of 308 AFP cases were inoculated on RD and L20B cell lines. Of these, 17 (5.5%) showed growth on both L20B and RD cells and 32 (10.4%) grew on RD cells only. All isolates that Showed CPE on L20B only were subjected to rRT-PCR using degenerate primers and probes from Centres of Disease Control and Prevention (CDC) which amplified the VP1 region according to CDC protocol [12]. No cases associated with wild poliovirus was found, all 28 isolates from 17 cases were characterised as Sabin-like polioviruses; 21 Sabin-like 1, 4 Sabin-like 2 and 2 Sabin-like 3 from 14, 2 and 1 AFP cases respectively. One sample was a mixture of poliovirus types 2 and 3. Further characterisation using non-degenerate primers and probe for rRT-PCR to determine the origin of the vaccine strains showed that none was vaccine-derived poliovirus (VDPV) but were all normal Sabin strains.

**Figure 1:**
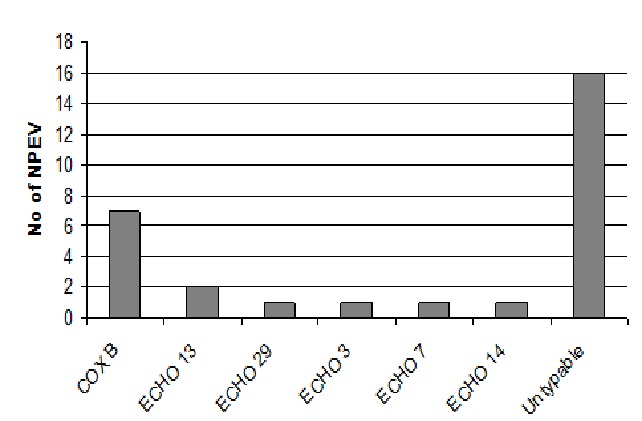
Types of non-polio enteroviruses implicated in AFP cases in Ghana in 2009

**Figure 2:**
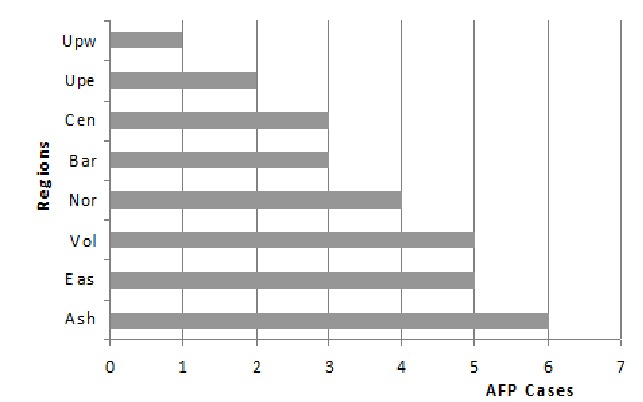
Geographical distribution of non-polio enteroviruses in Ghana, 2009

**Figure 3:**
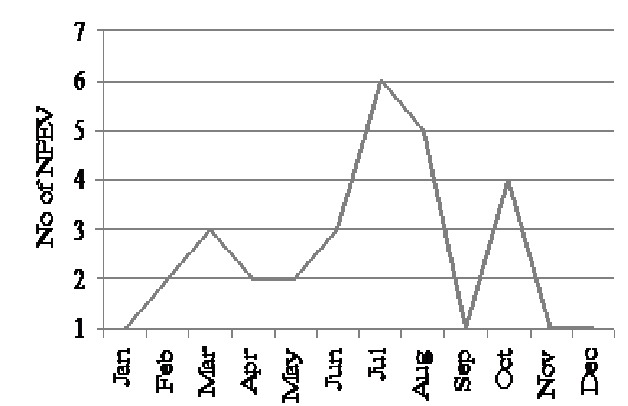
Seasonality of non-polio enterovirus in Ghana, 2009

Isolates that showed growth on RD cells only were identified by microneutralization using antisera pools raised against Coxsackie and echoviruses prepared by RIVM. As shown in Figure 1, six different serotypes of NPEVs were in circulation in 2009. Cox B viruses were predominant among AFP cases followed by Echo 13. However, majority of the isolates 17 (58.6%) were untypable by the antesera pools. The geographic distribution of the NPEVs isolated is shown in Figure 2. Non-polio enteroviruses circulated in 8 of the 10 region of Ghana in 2009. Distribution was however spread from the northern through the middle to the southern belts of the country. Ashanti region located in the middle belt saw more cases with the least from the Greater Accra region in the southern belt. Seasonal distribution revealed more NPEV in July and August after the raining season with the least around December and January during the dry season (Fig. 3).

The Expect Committee on Polio in Ghana meets quarterly to classify all AFP cases. As shown in Table 1, the Committee classified 308 cases out of which 33 were classified as compatibles. These were cases with two stools collected 24 hours apart within 14 days of onset of paralysis and therefore did not satisfy the criteria for stool adequacy. Annualize non-polio AFP rate of 2.58 cases per 100,000 children aged less than 15 years and stool adequacy of 75.0% were achieved. Twenty-five children were found with residual paralysis after 60-90 days of onset of paralysis. Three of these cases were as a result of non-polio enteroviruses; one Coxsackie virus and two untypable NPEV. One case of Sabin-like 1 poliovirus was found to be vaccine associated paralytic polio (VAPP) while no cause was found for the remaining 21 cases. The VAPP case was from a 4 year old child from the Brim Central district in the Eastern region. He had received 5 doses of OPV and had the date of onset on June 13, 2009. Stool was adequately collected and transported to the laboratory in good condition. Type 1 Sabin like poliovirus was isolated and the child had residual paralysis after 60 days of onset.

**Table 1:** Final classification of 2009 AFP cases by the Expect Committee on Polio

Indicator	Achievement
Number of AFP cases presented for classification	308
Number of cases compatible	33
Number discarded as non-polio AFP	273
Number lost to follow up	5
Number of deaths before follow up	2
Routine OPV (Oral Polio Vaccine) 3 coverage	89
National Immunization Days Round 1 (Feb 09)	99.7
National Immunization Days Round 2 (Mar 09)	103.5
National Immunization Days Round 3 (May 09)	104.1

## Discussion

The Global Polio Eradication Initiative has succeeded in reducing the circulation of wild poliovirus but non-polio enteroviruses implicated in AFP still remain a treat to the eradication initiative. In this study, feacal samples received from patients suspected of AFP were processed and characterised by rRT-PCR and antibodies to access the progress of polio eradication in Ghana since the last polio outbreak in 2008.

The findings confirmed 28 [9] cases of patients with AFP as Sabin-like polioviruses by rRT-PCR. All 3 serotypes of Sabin polioviruses were found with type-1 being the dominant. This is expected especially in countries with routine use of OPV or cluster around the time of NIDs. Unlike some countries [13] where vaccine strains have diverged significantly from the Sabin strain and have become vaccine-derived polioviruses as a result of several NIDs conducted, the Ghanaian isolates were normal Sabin polioviruses. Our findings further confirm that VDPV is rare since Ghana had used OPV in routine immunization, NIDs and sub-NIDs for 18 years.

Apart from the polioviruses, 29 NPEVs were also found with the isolation rate of 9.4%. This rate is lower than the expected rate of 10% and could be due to sample collection, storage and shipment to the laboratory. The results were also contrary to stydies elsewhere and may be connected to the small sample size but are typical of most tropical countries [6, 7]. The NPEV rate is useful indicator of laboratory performance but may be influenced by a number of factors, including the season of the year, elevation, or population hygienic levels. The NPEV circulated in 8 of the 10 regions of the country with the majority in Ashanti region. Furthermore, Coxsackie viruses that were predominant NPEVs in circulation were found more in Ashanti region than the other regions. The large number of unidentified NPEVs which could not be typed using the standard antisera contained in the kit is a limitation on the choice of assay and molecular methods should be used in further classification of these viruses. Aggregated forms of NPEVs, reoviruses or adenoviruses could also not be ruled out [7]. According to reports, serum raised against one serotype does not cross neutralize others, but serotypes can undergo some antigenic variation and limited neutralization is speculated to occur between several serotypes [12].

The peak of NPEVs’ infection was in the raining season between July and August and lowest in November to January, the dry harmattan period. In tropical and semitropical areas NPEVs circulation tends to be year round, or often associated with the rainy season, in temperate areas NPEVs were most prevalent in the summer and fall, although outbreaks may continue into the winter. This seasonal prevalence is typical of NPEVs worldwide, and this explains why they are commonly referred to as “summer viruses” in temperate countries [4]. Currently, there are no vaccines available for NPEVs, but as efforts is intensified on poliovirus eradication, there is the need to assess the impact of the disease burden caused by this group of viruses with a view to finding permanent solution where possible. It is speculated that as the global eradication of polioviruses approaches, proportionately more cases of NPEVs infections may emerge causing AFP and mimicking acute paralytic poliomyelitis [8].

Reports from the Polio Expect committee on 60-day follow-up classified all 308 cases as AFP of which 273 were discarded. These included all 28 Sabin-like polioviruses since samples were collected adequately with good laboratory conditions. One of the patients with AFP from whom the Sabin-like poliovirus type-1 was isolated was found with residual paralysis during the 60-day follow up. This is the first reported case of VAPP in Ghana even though some cases might have gone unnoticed. VAPP is clinically indistinguishable from paralytic poliomyelitis caused by wild-type polioviruses [15] and occurs among healthy OPV recipients and their contacts, with onset temporally linked (within 60 days) to OPV exposure. As the world approaches polio-free state, early detection and elimination of VAPP is a public health priority in Ghana. In the USA, polio vaccination policy was changed from OPV to exclusive IPV to eliminate VAPP cases [14].

Thirty-three cases were classified as compatible because samples were not collected adequately or did not reach the laboratory in good condition. Stool adequacy is an important indicator in polio surveillance as it enhances the chances of poliovirus isolation from stools. Increased number of compatible cases raises doubt on surveillance performance and the progress of polio elimination in a country. One of the 3 NPEVs isolated during the AFP surveillance that were found associated with residual paralysis was Cox B. This is consistent with other study reports where Cox B viruses were found with residual paralysis after 60 days of onset of paralysis [6, 7]. Furthermore, this study found 2 of the unidentified NPEVs associated with residual paralysis in children. Since factors that affect transmission of human enterovirus include overcrowding, levels of hygiene, water quality and sewage handling facilities [7], improved sanitation and general hygiene, provision of portable drinking water and discouraging overcrowding is vital to reducing the incidence of HEVs infections particularly among infants and children.

## Conclusion

Our study revealed that even though three of the non-polio enteroviruses and one Sabin-like poliovirus that circulated in 2009 left the children with paralysis, no wild polioviruses were detected. However, it is essential that Ghana continues to maintain high vaccination coverage and a sensitive surveillance system to rapidly detect and respond to cases of suspected paralytic poliomyelitis, from indigenous source, imported virus and from possible breaches in laboratory containment. Poliovirus from any of these sources into the Ghanaian communities with low vaccine coverage may result in endemic or epidemic transmission.
